# Rhythm perception is shared between audio and haptics

**DOI:** 10.1038/s41598-022-08152-w

**Published:** 2022-03-09

**Authors:** Corentin Bernard, Jocelyn Monnoyer, Michaël Wiertlewski, Sølvi Ystad

**Affiliations:** 1 CNRS, PRISM, Aix-Marseille Univ, Marseille, France; 2 Centre Technique de Vélizy, Stellantis, Paris, France; 3 CNRS, ISM, Aix-Marseille Univ, Marseille, France; 4 TU Delft, Delft, The Netherlands

**Keywords:** Touch receptors, Auditory system, Perception, Sensory processing

## Abstract

A surface texture is perceived through both the sound and vibrations produced while being explored by our fingers. Because of their common origin, both modalities have a strong influence on each other, particularly at above 60 Hz for which vibrotactile perception and pitch perception share common neural processes. However, whether the sensation of rhythm is shared between audio and haptic perception is still an open question. In this study, we show striking similarities between the audio and haptic perception of rhythmic changes, and demonstrate the interaction of both modalities below 60 Hz. Using a new surface-haptic device to synthesize arbitrary audio-haptic textures, psychophysical experiments demonstrate that the perception threshold curves of audio and haptic rhythmic gradients are the same. Moreover, multimodal integration occurs when audio and haptic rhythmic gradients are congruent. We propose a multimodal model of rhythm perception to explain these observations. These findings suggest that audio and haptic signals are likely to be processed by common neural mechanisms also for the perception of rhythm. They provide a framework for audio-haptic stimulus generation that is beneficial for nonverbal communication or modern human-machine interfaces.

## Introduction

When we explore a texture with our fingers, the interaction between the skin and the surface produces vibrations that propagate both through the air, up to our ears, and through our skin, down to our mechanoreceptors. Both sensory channels contribute to the perception of the texture properties^1^. These audio and tactile vibrations emanating from the same source are perceptually merged into a single amodal percept, creating a mental image of the surface^2^. As both stimuli share the same origin, the two modalities greatly influence each other. Altering the frequency content of the touch-produced sound can bias the perception of tactile roughness^3,4^. This effect, that can be produced when we rub our hands together, is known as the parchment skin illusion^5^. While psychophysical experiments demonstrate high-level interactions between audio and tactile sensory systems^6–9^, neuroimaging studies suggest that these interactions also occur in early sensory areas^10–12^. These experiments reveal strong interactions and common neural processes for vibrotactile perception and pitch perception, for frequencies above 60 Hz. However, audio-tactile interactions with lower frequency content, associated with rhythm, in particular rhythmic changes, are rarely investigated.

In the present paper, we investigated the perception of audio and haptic stimuli in which the rhythm evolves continuously with time. We decided to use the term *rhythm* that is here considered as the succession of events forming periodic patterns, which elements are distinguishable from each other, sticking to the definition given by Cooper et al.^13^: “to experience rhythm is to group separated sounds into structured patterns”. We use the term for beat rates up to 60 Hz^14^, frequency range that is more commonly characterized as flutter range in tactile perception^15^. Whether the sensation of accelerating or decelerating rhythms is shared between audio and haptic perception remains unknown. In audio, these evolving stimuli are better known as accelerando or decelerando, in the case of tempo increase or decrease. In touch, it has been shown that a 10% variation in the ridge density can be detected^16,17^. Here, we generated haptic stimuli whose spatial frequency gradually evolves during exploration by a finger on a glass plate actuated with ultrasonic friction modulation. This method uses ultrasonic levitation to change the friction between the finger and the glass^18^. Modulating friction in reaction to users’ exploratory motion produces sensations of texture, shape and relief on a flat surface^19–21^. In addition, the use of synthesized stimuli makes it possible to freely combine auditory and haptic stimuli. A similar setup has already been used to show audio-haptic perception changes with aging^22^.

**Figure 1:**
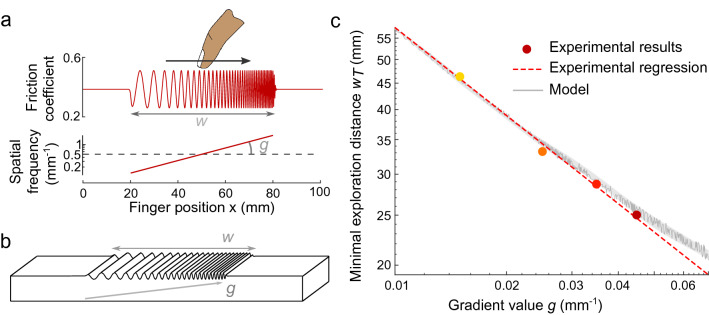
Overview of the experiment on haptic gradient detection thresholds. Subjects explore a synthetic sinusoidal grating whose spatial frequency evolves exponentially with respect to the finger position. **a.** Illustration of the haptic stimulus for the gradient value condition $g=0.045~{\mathrm {mm}}^{\mathrm {-1}}$ and the window size $w=60$ mm. This stimulus is the one with the widest spatial frequency range, from $\nu = 0.13$ to $1.93~{\mathrm {mm}}^{\mathrm {-1}}$ with a central frequency $\nu _0=0.5~{\mathrm {mm}}^{\mathrm {-1}}$. Explored with a finger velocity of approximately $v=60$ mm/s, it produces vibration of frequency $f=\nu *v$ from 8 to 116 Hz, centered on 30 Hz. Stimuli at the thresholds stay within 17 to 52 Hz. **b.** Illustration of the illusion produced by modulating fingertip friction with the haptic interface. **c.** Minimal exploration distances $w_T$ necessary to detect the variation in ridge density, shown as dots for various gradient values *g*. Logarithmic regression, shown by the dashed red line, leads to a goodness of fit $R^2=0.997$. The model predictions are shown in dark gray, and the margin of error is shown in light gray.

In the present study, we modulated the friction with respect to the position of the user’ finger. The modulation is a spatial sinusoidal wave, which spatial frequency gradually increases or decreases, becoming finer or coarser. This process is illustrated in Fig. 1b. Touching these haptic stimuli produces the sensation of bumps that becomes closer or more distant from each other, like accelerating or decelerating rhythmic patterns.

The perception of these haptic gradients is here investigated by a psychophysical experiment, whose results are compared with the literature on auditory perception. We further explain these observations with a multimodal model of rhythm perception. This model predicts similar auditory and haptic mechanism in the perception of rhythmic gradients, confirmed by a final multimodal experiment that demonstrates interaction between the two modalities.

## Results

### Haptic detection of periodicity changes

The longer a participant explores a texture, the better they are at discriminating if the frequency is increasing or decreasing. How fast they can detect the trend is a clear indication of their perceptual threshold. The first experiment investigates how the exploration distance influences the detection of gradient *g*, by constraining the exploration by a window *w*. The experimental design draws inspiration from studies on auditory perception, which explore the minimal duration needed to perceive a frequency or tempo change at a given rate of change^23–31^.

The detection thresholds were investigated for 4 gradient value conditions ($g=$ 0.015, 0.025, 0.035, 0.045 ${\mathrm {mm}}^{\mathrm {-1}}$) and 2 directions (increasing or decreasing) with 6 window sizes ($w=$ 10, 20, 30, 40, 50, 60 mm). A stimulus corresponding to the increasing direction is presented in Fig. 1a. In each trial, subjects were asked to synchronize their movement with a cursor to ensure a constant finger velocity. After exploring the stimulus once, they had to report if they felt that the stimulus “became finer” or “became coarser”, which corresponded to increasing or decreasing spatial frequencies, respectively. Subjects’ responses and the related analyses are presented in Fig. 7a,b in the Materials and methods section. The percentages of correct answers for all subjects and for each condition are fitted with psychometric curves to obtain the window size thresholds $w_T$. The minimal exploration distances to perceive a change in the gradient value g = 0.015, 0.025, 0.035 and 0.045 ${\mathrm {mm}}^{\mathrm {-1}}$ were found to be $w_t$ = 46.2, 33.1, 28.7 and 25 mm, respectively. The thresholds $w_T$ decrease as the gradient value *g* increases with a linear dependency on a logarithmic scale, as illustrated Fig. 1c. A logarithmic regression reveals a significant correlation (p=0.004) between the window size threshold and the gradient value such that $\log (w_T) = 1.50 - 0.55 \log (g)$, which can also be written as $w_T \times g^{0.55} = 4.48$.

### Comparison of audio and haptic thresholds

**Figure 2:**
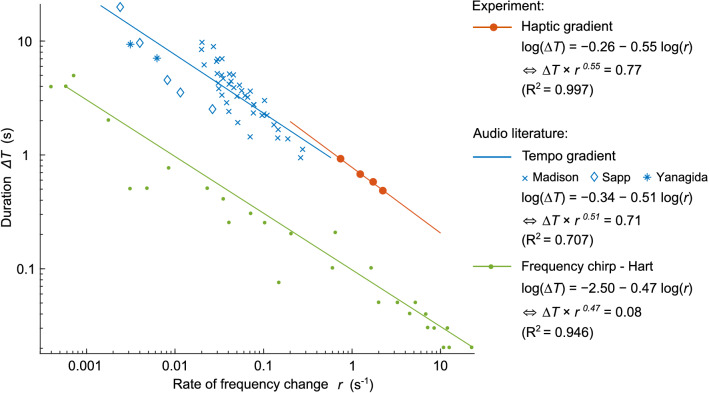
Comparison of the experimental results with the literature. Data points are fitted with logarithmic regressions (displayed as solid lines). The regression equations are presented in two forms with their goodness of fit. Thresholds for the haptic gradients, converted to time with the finger velocity value, are shown in red. Thresholds from the literature on the perception of tempo gradients (accelerando)^23–25^ are displayed in blue. Thresholds from the meta-analysis of Hart^26^, who gathered data from many studies on the perception of frequency chirps (glissando), are shown in green^27–31^.

The exploration distance $w_T$ and the gradient value *g* are not proportional, but follow a power law with an exponent of 0.55. To compare the results of this experiment with data from the literature on tempo and frequency gradients in auditory stimuli^23–31^, the exploration distances *w* (in mm) and gradient value *g* (in ${\mathrm {mm}}^{\mathrm {-1}}$) were converted into stimulus durations $\Delta T = w / v_{finger}$ (in s) and rate of frequency change $r=g \times v_{finger}$ (in $\mathrm {s^{-1}}$), respectively, using the finger velocity $v_{finger}=59.6\pm 9.7~\mathrm {mm/s}$. Participants were asked to explore the stimuli with a constant speed by synchronizing their finger movement with a cursor on a visual display. Figure 2 provides a comparison between our results and the literature data. The haptic gradient threshold curves strongly resemble the audio tempo gradient threshold curves, with the only difference being that the haptic thresholds are presented for shorter durations. Textures of a few centimeters explored at a velocity of approximately $50~\mathrm {mm/s}$ typically lasted approximately 1 s, which is indeed below the usual tempo durations for audio stimuli. The graph also shows that the slope distribution of the tempo and haptic gradients is close to that obtained for frequency chirps.

To numerically investigate these similarities, we performed logarithmic regressions, which yielded the equations in Fig. 2. We can compare the values of the exponent *e* and the constant *c* of the threshold laws $\Delta T \times r^{e} = c$. This analysis confirms that the 3 exponent values are in the same range and, most importantly, that the haptic and auditory tempo values differ by only 7.3% and 7.8% (relative error) for *e* and *c*, respectively.

In summary, haptic gradient thresholds follow the same law as rhythmic gradients. This suggests that similar mechanisms are activated in the two modalities for low-frequency gradient perception.

### Perceptual model of audio-haptic rhythmic gradients

To investigate these mechanisms, we adapted a model from the literature on the perception of irregular rhythmic patterns based on the work of Schulze^32^. Three theories compete to explain the encoding of tempo perception.The *successive interval discrimination* theory proposes that each interval between two beats is compared with the previous interval. When a difference exceeds a given threshold, an irregularity is perceived. Comparison with the *internal rhythm* theory states that the first beats are internalized and used as a rhythmic reference. When a beat differs by more than a threshold from the reference, an irregularity is perceived. Finally, the third theory, the *internal interval theory*, is similar to the *successive interval discrimination theory*, but uses the interval rather than the rhythmic difference. It postulates that the first interval is internalized and used as a reference. When a duration difference between one interval and the reference exceeds a certain threshold, an irregularity is perceived.

These theories were tested by Schulze on beat sequences that contained carefully chosen irregularities. The results of his study revealed that the *internal rhythm* theory was a good predictor, but that the results were also in agreement with the internal interval model predictions. The experiment was reproduced by Keele et al.^33^, who concluded that the *comparison with the internal interval* theory was more likely to predict the perceived rhythm. A generalization of Schulze’s model was later proposed to take into account the influence of the initial pace^34^. Investigating the perception of linear tempo gradients^23^, Madison explained his results using models of previous studies with the principle of accretion, in which the accumulation of small differences reinforces the global difference.

**Figure 3:**
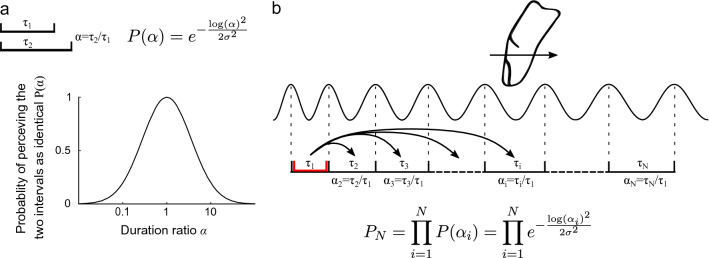
Model of tempo perception applied to haptics. (**a**) Basic principle of the model: the probability *P* of perceiving two intervals as having the same duration follows a log-Gaussian function with respect to the ratio between the two interval durations $\alpha$. (**b**) Application of the model: the haptic stimulus is converted into a pulse train. The theory of *comparison with an internal interval with accretion* is then applied to the intervals $\tau _i$ to calculate the probability of perceiving the gradient.

The haptic frequency gradient perception model, derived from its audio counterpart, is illustrated in Fig. 3. First, the haptic signal encoding the friction is converted into a pulse train, where each pulse corresponds to the local maximum of the virtual shape. The pulse train signal mimics the response of the Pacinian channels to sinusoidal stimulation^35^. The duration between two pulses $\tau$ is then computed. The probability of perceiving 2 intervals of duration $\tau _1$ and $\tau _2$ as identical is described by the probability distribution $P(\alpha )$. We assume that the probability of perceiving a difference in successive intervals depends on the duration ratio $\alpha =\tau _2/\tau _1$ and follows a log-normal function as presented in Fig. 3a. The standard deviation of logarithmic values $\sigma$ is the only fixed parameter in the model. We compared three theories of tempo perception (see Fig. 8 in the Materials and methods section). Among the three theories, the internal interval with accretion theory is the best predictor of the observed results. In this model, the first interval is internalized and used as a reference, and then each interval duration is compared to the reference duration to calculate the probability *P* of perceiving no difference. The small, imperceptible variations are compounded using the accretion principle and their accumulation reinforces the global difference. The overall probability of perceiving no change in the stimulus $P_N$ is then the product of all the previous probabilities $P(\alpha _i)$. The final probability of perceiving the change in frequency is given by $P_{g,w}=1-P_N$.

In line with the experimental design, this procedure is applied to all gradient magnitude *g* and window size *w* conditions. The theoretical thresholds are calculated by performing the same analysis with psychometric curve fittings (see Fig. 9 in the Materials and methods section). These thresholds are defined as the critical window sizes $w_{T}$ that yield a 50% chance of perceiving the irregularity ($P_{g,w_{T}}=0.5$). To minimize the error between the four $w_{T}$ values of the model and of the experiment, we optimize the parameter $\sigma$ of the log-normal distribution. We find that $\sigma = 1.153$ leads to the best predictions of the observed data, with a mean relative error of 1.74%. The proposed model can also extrapolate the experimental thresholds to a broader range of values, as presented in Fig. 1c.

### Audio-haptic interaction

**Figure 4:**
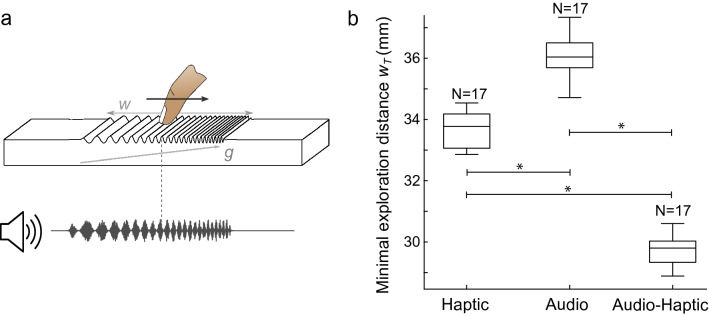
Audio-haptic interaction. (**a**) Illustration of the multimodal experiment with audio feedback derived from the haptic texture. (**b**) Perception threshold for the unimodal and multimodal conditions. The thresholds are represented by the minimal exploration distance to perceive a difference in frequency with the gradient value $g=0.025~{\mathrm {mm}}^{\mathrm {-1}}$.

The previous results hint at a shared process between the haptic and the audio perception of rhythmic gradients. To test whether both modalities do indeed influence each other, we measured their influence on the overall detection threshold when both modalities were present. This methodology was successfully used in previous studies to unravel the interaction between haptics and other modalities^36–38^. To detect a multimodal interaction, haptic stimuli were enhanced with congruent auditory stimuli also based on the finger movement. Audio feedback was synthesized from filtered white noise, which evokes a natural interaction sound, such as rubbing^39^. These signals were amplitude-modulated by an oscillator whose frequency matches that of the haptic stimuli used to render the virtual shape. The auditory stimuli were hereby perceived as successive beats with increasing or decreasing tempos perfectly synchronized with the haptic stimuli both in terms of modulation and time window, as illustrated in Fig 4a. Since the auditory stimuli were amplitude modulated signals derived from filtered white noise, no noticeable pitch was perceived, even for frequencies above 30 Hz .

In a second experiment, we investigated whether the addition of audio feedback could improve the gradient perception and thus lower the minimal exploration distance. Thresholds were investigated for the 0.025 ${\mathrm {mm}}^{\mathrm {-1}}$ gradient value condition with the same 6 window sizes (from 10 to 60 mm) for 3 modality conditions: haptic only, audio only and bimodal audio-haptic. For the audio only condition, subjects performed the same movement with their finger on the plate actuated with a constant friction level (50% of modulation) and then felt a flat surface. The analysis of the subject responses was achieved with the same algorithm as in the first experiment. We used the jackknife resampling method for the statistical analysis. This method is presented in the Materials and methods section, and the thresholds are presented in the boxplot in Fig. 4b.

A non-parametric Kruskal–Wallis test was performed with the sensory modality as a factor (haptic, audio or audio-haptic) on the 17 samples from the jackknife method. The test shows a significant effect (at $\alpha = 0.05)$ of the modality ($\chi _{2}^2 = 44 , p=2.2\times 10^{-10}$). This result was validated by post-hoc Nemenyi tests, which presented significant differences between the 3 modality conditions (H-A: $p=2.5\times 10^{-3}$, H-AH: $p=2.5\times 10^{-3}$, A-AH: $p=7.8\times 10^{-11}$).

Haptic-only and audio-only thresholds fall into the same order of magnitude, as we expected from the previous comparison in Fig. 2. However, when both modalities are combined, the detection threshold is significantly reduced. The two modalities contribute to heightening of the sensitivity, indicating a multimodal integration of rhythm.

## Discussion

Audio and haptic perception are known to interact for pitches above 100 Hz, and in this series of experiments we demonstrated that this interaction extends to the perception of rhythm and its temporal evolution. When exploring with our bare finger a simulated surface, rhythm was haptically perceived after a minimal exploration distance. This distance followed a power law with the rate of change in the frequency, with an exponent of $\approx 0.5$, matching the known behavior of auditory signal perception when the tempo increases or decreases. A model of audio tempo perception, based on an internal interval theory, accurately predicted the results of the haptic experiment.

Adding congruent audio feedback to the haptic sensation resulted in a significant interaction between the two modalities. When the auditory variations of the tempo followed the shape of the haptic signal, the participant needed 12% less distance to achieve an accurate detection. These results attest to a bimodal integration of audio and haptic stimuli, which suggests that the perception of the energy envelope of audio and haptic signals shares common perceptual mechanisms.

The model predicts the probability of perceiving both audio and haptic rhythmic variations on a wide range of variation rates and durations; however, its applicability has limits. If we take a closer look at the probability distribution shown in Fig 3a, it is difficult to interpret the value of the parameter $\sigma = 1.153$. This value would lead to a just noticeable difference between two interval durations of 289% (P(0.389)=0.5), which is not coherent with the just noticeable difference of $\approx 10\%$ reported in previous works^40^.

Because of the protocol design, some stimuli in our experiment tended to exceed the flutter range (<60 Hz) at their extremity, making the interpretation more complex. However, this issue concerns only the stimuli with the longest time windows; the stimuli at the thresholds all remain within 17 to 52 Hz, corresponding to the flutter range. This limit comes from the fact that two tactile events need to be separated by at least a certain duration to be perceived independently. Considering two successive isolated pulses, the minimal interval is about 40 ms^41^, whereas with pulse trains, the limit between the flutter range (discrete events) and the continuous vibration range was evaluated at 60 Hz ( $\sim$15 ms intervals)^15,42^. This value has been verified using both periodic^43,44^ and aperiodic^45^ stimuli: the authors showed that the tactile sensation of frequency is determined by the duration of the silence intervals between the pulses. Intervals longer than 15 ms are crucial whereas shorter intervals have only a limited effect on the frequency evaluation, exhibiting the limit of the discrete perception below this value. Haptic signals with higher frequencies are felt more as continuous textures. This principle also occurs in audition: audio beating progressively turns into sound roughness from 30 to 100 Hz, and then to pitch. The present model is limited to a specific frequency bandwidth, and does not take into account phenomena that occur outside this band. However, the comparison principle and accretion of the probabilities are likely to be effective for the perception of higher frequency changes as well, as seen in Fig. 2. In the future, this limit of the model could be overcome by applying weights to each interval with respect to its length to minimize the effect of small indistinguishable intervals, as the approach discussed previously^43,45^.

The findings extend previous works that showed similarities between the perception of tactile vibration and auditory pitch to the perception of discrete, dynamic low-frequency stimuli: time-varying haptic gratings and audio pulse trains. In addition, the results are of interest in the field of human-machine interfaces for the design of textural haptic feedback to guide the user on touchscreens without requiring visual attention^46^. Other studies have shown that haptic gradients with intensity variations are promising for guiding exploratory motion^47,48^. Our findings extend this promise by showing an unambiguous relationship between the exploration window and the magnitude of the gradient ($w \times g^{0.55} > 4.48$ ) to create salient stimuli. The perception of these stimuli can be further enhanced by adding congruent audio feedback.

In addition, these results open up new perspectives related to nonverbal communication and sensory substitution. In speech, for instance, frequency transitions are central in conveying information^30^. The emotional aspect of speech is strongly conveyed through fundamental frequency changes (f0 trajectory) of voiced vowels (parent-child communication)^49^. It has been shown that downward pitches are often associated with cold or anger and rising pitches with fear, surprise and happiness^50^. Both temporal and frequency variations (portamento, accelerando/ritardando) are also extremely important for conveying emotions through music^51^. Hence, although only time-varying pulses have been explored in the present study, perceived glissandi in the haptic domain could help produce emotional reactions in line with musical stimuli, since similar mechanisms are activated in the tactile and auditory domains.

## Methods

### Haptic gradient construction

Haptic gradients are spatially encoded signals, in which the spatial frequency $\nu$ (in ${\mathrm {mm}}^{\mathrm {-1}}$) evolves as a finger explores a surface. The spatial frequency can be considered as the number of ridges per millimeter. For the gradient evolution to be perceived equally along the frequency range, we adapted the spatial frequency to the Weber Law. According to this law, the just noticeable difference (JND) $\delta \nu$ of the frequency is proportional to the initial frequency $\nu$ multiplied by a constant of proportionality *g*:

1 $$\begin{aligned} \delta \nu =g\nu \end{aligned}$$

We called *g* the gradient value (in ${\mathrm {mm}}^{\mathrm {-1}}$). The instantaneous spatial frequency of the grating $\nu$ obtained by integrating $\nu$ varies as a function of the finger position *x* (in mm) according to the following relationship:

2 $$\begin{aligned} \nu (x)=\nu _{0} \exp (g(x-x_0)) \end{aligned}$$

where $\nu _{0}=0.5~{\mathrm {mm}}^{\mathrm {-1}}$ is the central spatial frequency, which is the same for all stimuli, *x* is the finger position and $x_0=50$ mm is the center of the glass plate. The sine wave gradient $y_g$ that oscillates at the instantaneous frequency $\nu$ was then defined as follows:

3 $$\begin{aligned} y_g(x)=\cos \left( 2\pi \int _{0}^{x} \nu (u)\, du \right) \end{aligned}$$

Thus, to avoid potential influences due to perceived intensity variations, the stimulus amplitude was adjusted under the 50 mm/s finger velocity condition according to data from a previous experiment^52^ that provided frequency-dependent intensity judgments obtained with the same haptic device as in the current study. This adjustment represents a corrective factor $a_c(\nu )\in [0.5,~1]$, which attenuates the signal in the mid-frequency bandwidth. A maximum attenuation of 0.5 was hereby applied at $\nu =2~{\mathrm {mm}}^{\mathrm {-1}}$. The intensity of the stimulus was therefore perceived as constant along the gradient.

The windowing function $\phi$ for a given window size *w* (in mm) was defined as the difference between two sigmoidal functions:

4 $$\begin{aligned} \phi (x) =\frac{1}{1+ e^{-5(x-x_0 + w/2)}} - \frac{1}{1+ e^{-5(x-x_0 - w/2)}} \end{aligned}$$

The windowing function was also centered on $x_0$. Finally, the resulting signal of the stimulus *A*(*x*) with respect to the finger position was given by:

5 $$\begin{aligned} A(x) = \frac{1}{2} + \frac{1}{2}\phi (x) a_c y_g(x) \end{aligned}$$

**Figure 5:**
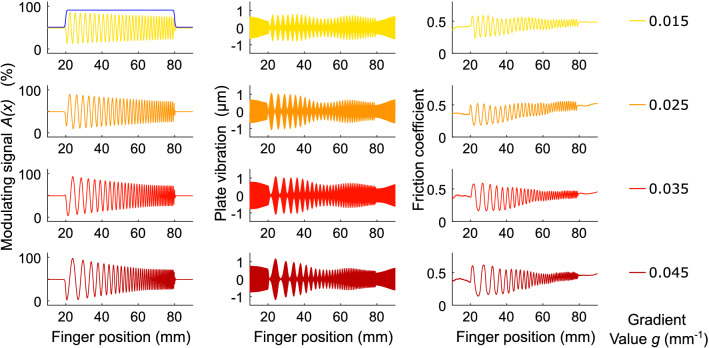
Presentation of the stimuli for the 4 gradient values (from top to bottom of the figure) under the 60 mm window condition with 3 types of measures. The left figures present the amplitude command of the modulating signal, the center present the measured vibration of the glass plate and the right present the measured friction coefficient between the finger and the surface. The amplitude is attenuated for higher frequencies to equalize the perceived intensity of the stimuli. Friction measurements are performed with a sensor described in a previous work^52^.

A(x) is the modulating signal (in %) encoding the friction, which is electronically multiplied by the ultrasonic carrier signal. It is presented for the 4 gradient value conditions and the 60 mm window size condition in Fig. 5.

### Apparatus

In this setup, ultrasonic friction modulation is achieved on a $\mathrm {105 \times 22 \times 3.3~mm}$ glass plate. To track the finger position of the subject, a small ring is attached on the first phalanx of his/her index finger, which is connected to a pulley-encoder system. It measures unidirectional displacements along the length of the glass plate with an accuracy of approximately 0.01 mm and a refresh rate of 4 kHz without any significant latency. A microcontroller (Teensy 3.5) reads the encoder and outputs a modulating signal (first column of Fig. 5) according to a friction map corresponding to the haptic stimulus. The carrier signal, a 35 kHz sine wave, is created by a function generator (BK Precision 4052) and amplitude-modulated by the analog signal provided by the microcontroller. The resulting signal is then amplified 20-fold (WMA-100, Falco Systems) to drive two piezoelectric actuators glued to the glass plate. Modulation of the amplitude of vibration of the glass plate (second column of Fig. 5) induces friction variations between the finger and the plate during the tactile exploration (third column of Fig. 5).

The graphical interface of the experiment, made with Max/MSP, is connected to the microcontroller with serial communication. It handles the subjects’ responses and audio feedback for the second experiment by receiving the finger velocity $v_{finger}$ (approximately 50 mm/s), spatial frequency $\nu$ and windowing values in real time. The audio feedback is constructed as follows: filtered white noise (Butterworth 2nd-order bandpass filter between 400 and 800 Hz) is modulated from 0 to 100% by an oscillator at a frequency $f=\nu *v_{finger}$. Then, the windowing value acts as a gain from 0 when the finger is outside the window to 1 when the finger is on the haptic stimulus. Since the modulation signal and the time window are managed by the microcontroller, based on the finger position, for both audio and haptic actuation, the stimuli for both modalities are perfectly synchronized. The only delay that comes from the serial communication between the microcontroller and the Max/MSP sound generator on the computer is not noticeable. Sounds are played through headphones (Sennheiser HD 26 Pro).

### Protocol

**Figure 6:**
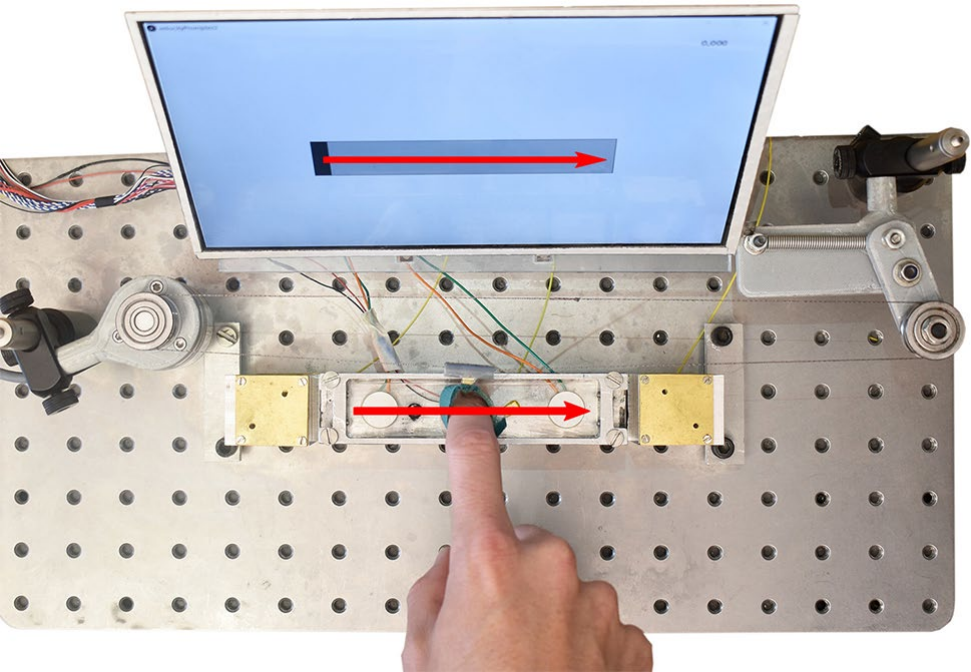
Experimental setup. The subject touches the actuated glass plate from left to right. The finger is linked to a pulley-encoder system for position measurement. The screen shows a cursor imposing the finger velocity.

Participants sat in a chair in front of the experimental desk and attached the ring connected to the position-tracking apparatus to their right index finger. Headphones prevented any external auditory cues from the device. In each trial, the participants were asked to explore the actuated glass plate by sliding their finger from left to right and to synchronize their movement with a cursor presented on a screen in front of them that imposed a finger velocity of 50 mm/s, as presented in Fig 6. Participants could explore the stimulus only once. They were then asked to determine whether they felt that the ridge density increased (toward a “finer” texture) or decreased (toward a “coarser” texture) via a keyboard on the left-hand side of the setup. A training session familiarized the subjects with the terms and the corresponding stimuli.

In the first experiment, gradient perception was investigated following the method of constant stimuli for 4 value and 2 direction conditions, i.e., $g=\pm 0.015,~\pm 0.025,~\pm 0.035,~\pm 0.045$ ${\mathrm {mm}}^{\mathrm {-1}}$, 6 window size conditions, i.e., $w=$ 10, 20, 30, 40, 50, 60 mm, and 8 repetitions, which led to $4 \times 2 \times 6 \times 8 = 384$ trials. Stimuli were presented in random order. Other parameters, such as the finger velocity and the central frequency of the gradient, were set as constants.

During the second experiment on multimodal perception, audio feedback was delivered through the headphones. The protocol was almost the same: 3 modality conditions, i.e., haptic only, audio only, and audio-haptic, for one gradient value and 2 directions, i.e., -0.025 and +0.025 ${\mathrm {mm}}^{\mathrm {-1}}$, 6 window size conditions, i.e., 10, 20, 30, 40, 50, 60 mm, and 8 repetitions, which led to $3 \times 2 \times 6 \times 8 = 288$ trials. The whole experiment lasted for approximately 90 minutes.

### Subjects

Twenty-one subjects (5 females), 20 right-handed and 1 left-handed, ranging from 19 to 52 years old (mean 29) participated in the study. All the subjects participated in both experiments. They gave their informed consent before the experiment. All procedures were approved by the Ethical Committee of Aix-Marseille University and the experiment was carried out according to the relevant guidelines and regulations expressed in the 1964 Declaration of Helsinki. They were paid for their participation. They washed and dried their hands before the experiment, and the glass plate was regularly cleaned with an alcoholic solution. Three subjects showed incoherent results, either due to technical issues or misunderstanding of the task. We defined a criterion for subject exclusion based on the percentage of correct answers under the easiest conditions: $g=0.045~{\mathrm {mm}}^{\mathrm {-1}}$ and $w = 50$ and 60 $\mathrm {mm}$. The three subjects appeared as outliers according to the Tukey Fences method applied to these criteria, and their results were therefore discarded. Concerning the multimodal experiment, the same criterion was applied to the audio condition, and another subject was classified as an outlier. This subject’s results were discarded from the multimodal analysis only.

### Threshold measurement and statistical analysis

**Figure 7:**
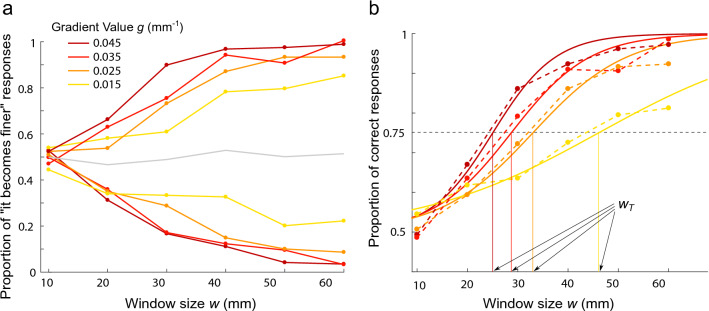
(

For each window size and gradient value condition, the answers from all the subjects were gathered to calculate the proportion of trials in which the stimulus was felt as becoming finer or coarser. The results, presented in Fig. 7a, reveal that for the smallest window size condition (10 mm), the subjects were not able to feel the difference between increasing and decreasing gradients, but this difference became more perceptible as the window size increased.

Since the mean of the proportions across gradient value conditions (gray line) remained at approximately 0.5, there was no bias toward one type of response. It was therefore possible to average the increasing and decreasing conditions to obtain a common (direction-independent) gradient value. The proportion of correct answers calculated accordingly is presented in Fig. 7b. Similarly, we can observe that for small window size conditions, the proportion of correct answers was around the level of pure (50%), and as the window size increased, subjects tended to achieve correct answers 100% of the time, with slight variations within gradient value conditions. These data were fitted by psychometric curves given by the sigmoid function with the parameters $\gamma$ and $\beta$:

6 $$\begin{aligned} sig(w) =0.5 + \frac{0.5}{1+ e^{-\gamma (w-\beta )}} \end{aligned}$$

The psychometric curves enabled us to measure the exploration distance thresholds required to perceive the gradient, i.e., the minimal exploration windows $w_T$ to obtain 75% correct answers. $w_T$ for the 4 gradient value conditions was calculated from the results of all 18 subjects. To measure if the effect of the gradient value was significant, we performed a method based on the jackknife resampling technique^53^ used in^54^ and^55^. This method, also called “leave-one-out”, consists of running the analysis repeatedly while excluding one of the 18 subjects for each run, which means that the operation was repeated 18 times. A nonparametric Kruskal–Wallis test was performed on the 18 samples with the gradient value as factor. The test revealed a significant effect (at $\alpha = 0.05)$ of the gradient value ($\chi _{3}^2 = 67 , p=2.3e^{-14}$). For the second experiment on multimodal integration, we ran exact same analysis procedure.

### Comparison of models with different theories

**Figure 8:**
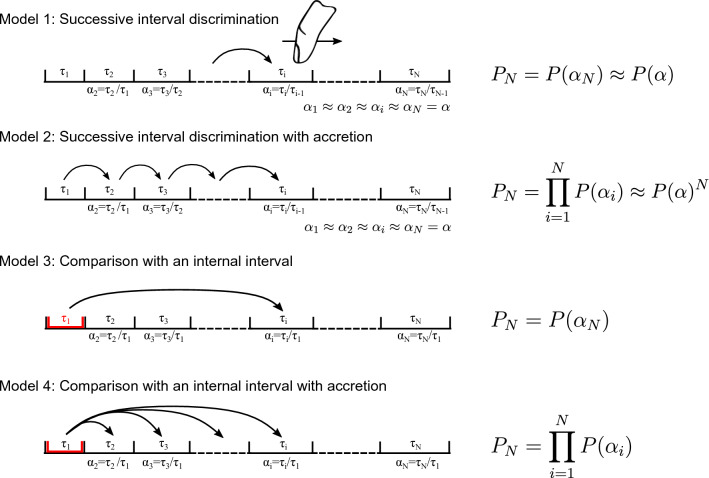
Presentation of the models performed with 4 theories on tempo perception adapted from the literature.

The model we proposed was also evaluated with alternative theories of tempo perception derived from the literature, as presented in Fig. 8.

1. Successive interval discrimination: each interval is compared with the previous interval.
2. Successive interval discrimination with accretion: each interval is compared with the previous interval and previous comparisons are kept in mind.
3. Comparison with an internal interval: the first interval is internalized, and each interval is compared with this reference.
4. Comparison with an internal interval with accretion: the first interval is internalized, and each interval is compared with this reference. Previous comparisons are kept in mind. This is the model presented in the core of the article.

Because the stimuli of the experiment present intervals that are either all increasing or all decreasing, there are no irregularities due to variation in the direction. Hence, we do not evaluate the theory of comparison with an internal rhythm from^32^. In our case, this is equivalent to the theory of comparison with an internal interval.

**Figure 9:**
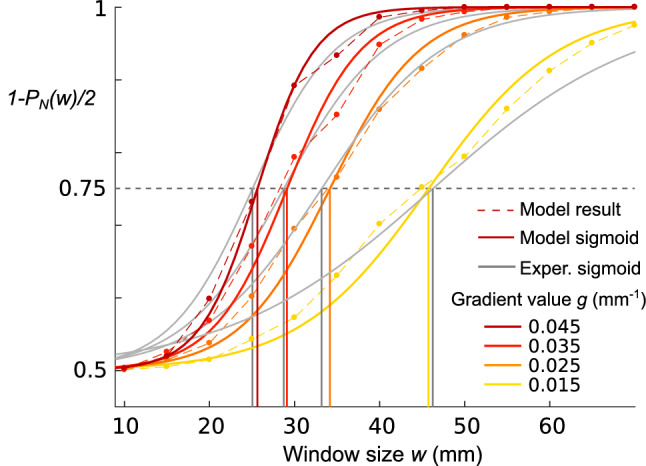
Comparison between the prediction of Model 4 and the experimental results. Probabilities are calculated with $\sigma _{opt}=1.153$. The probability of perceiving the gradient variation is plotted for window sizes every 5 mm for the 4 gradient value conditions. These data are fitted by sigmoid curves to find the window size thresholds predicted by the model. Thresholds from the experiment are plotted in gray.

For a given gradient value *g* and window size *w*, the corresponding haptic stimulus is converted into a pulse train, where each pulse coincides with a maximal friction value. According to the interval duration of the pulse train, the probability of not perceiving any change $P_N$ is computed. These probabilities are plotted in Fig. 9. The value $1-P_N/2$ is preferred to match the experiment, which is designed with a two stimuli-one interval paradigm. The analysis is then the same as for the experimental results. Data are fitted with psychometric curves to measure the window size which gives $1-P_N/2 = 0.75$.

For each model, an optimization of the parameter of the probability distribution function $\sigma$ (see Fig. 3a) is performed. A gradient descent algorithm finds the value of $\sigma _{opt}$ that minimizes the root-mean-square error between the predicted thresholds $w_T$ from the model and those from the experiment. Table 1 compares the accuracy of the models.

**Table 1:** Results of the optimization of $\sigma$ on the root-mean-square error (RMSE) between the predicted thresholds and the thresholds from the experiment for the 4 models.

	$\sigma _{opt}$	RMSE	error %
Model 1	/	/	/
Model 2	0.118	90.06	30.32
Model 3	0.539	13.71	11.04
Model 4	1.153	0.412	1.74

The error is also expressed as the percentage error for visualization. Optimization with model 1 is not possible.

Because $P_N$ does not change with the number of intervals *N* for model 1, since the window size has no influence on $P_N$, optimization is not possible. This model is thus discarded. Comparing the errors, it appears unequivocally that model 4 has the best prediction performance based on the experimental results. This model is also the one that stands out from the literature.
